# A broad-spectrum and highly potent human monoclonal antibody cocktail for rabies prophylaxis

**DOI:** 10.1371/journal.pone.0256779

**Published:** 2021-09-01

**Authors:** Pan Kyeom Kim, Jung Sun Ahn, Cheol Min Kim, Ji Min Seo, Sun Ju Keum, Hyun Joo Lee, Min Joo Choo, Min Soo Kim, Jun Young Lee, Ki Eun Maeng, Ji Young Shin, Kye Sook Yi, Modupe O. V. Osinubi, Richard Franka, Lauren Greenberg, Madhusudana Shampur, Charles E. Rupprecht, Soo Young Lee

**Affiliations:** 1 Department of Research and Development, Celltrion, INC, Incheon, Republic of Korea; 2 Centers for Disease Control and Prevention, Atlanta, Georgia, United States of America; 3 National Institute of Mental Health and Neurosciences (NIMHANS), Bangalore, India; 4 LYSSA LLC, Cummings, Georgia, United States of America; University of Pretoria, SOUTH AFRICA

## Abstract

Post-exposure prophylaxis (PEP) is highly effective in preventing disease progression of rabies when used in timely and appropriate manner. The key treatment for PEP is infiltration of rabies immune globulin (RIG) into lesion site after bite exposure, besides wound care and vaccination. Unfortunately, however, RIG is expensive and its supply is limited. Currently, several anti-rabies virus monoclonal antibody (mAb) products are under development as alternatives to RIG, and two recently received regulatory approval in India. In this study, fully human mAbs that recognize different rabies virus glycoprotein conformational antigenic site (II and III) were created from peripheral blood mononuclear cells of heathy vaccinated subjects. These mAbs neutralized a diverse range of lyssavirus types. As at least two anti-rabies virus mAbs are recommended for use in human PEP to ensure broad coverage against diverse lyssaviruses and to minimize possible escape variants, two most potent mAbs, NP-19-9 and 11B6, were selected to be used as cocktail treatment. These two mAbs were broadly reactive to different types of lyssaviruses isolates, and were shown to have no interference with each other. These results suggest that NP-19-9 and 11B6 are potent candidates to be used for PEP, suggesting further studies involving clinical studies in human.

## Introduction

Rabies is a major viral zoonosis that remains a significant and neglected global public health problem [1–5]. This acute, progressive, incurable encephalitis disproportionately affects rural and underserved communities, especially low- and middle- income countries (LMICs), where canine rabies is poorly controlled and access to appropriate medical care is limited or non-existent [6]. Without intervention prior to disease progression, rabies has the highest case fatality of any infectious disease [7, 8]. Globally, rabies is responsible for tens of thousands of human deaths annually, with rabies infection from dogs accounting for over 99% of the cases [9–11]. Post-exposure prophylaxis (PEP) for individuals with suspected rabies exposure is effective when appropriately administered in a timely manner [12]. Besides wound washing and administration of modern cell culture vaccines, infiltration of rabies immune globulins (RIG) to bite lesions site provides immediate passive immunity until the active induction of virus neutralizing antibodies (VNA) from vaccination [13–17]. When administered according to guidelines, the efficacy of PEP in preventing disease progression is nearly 100%. Despite its critical utility as an essential medicine for PEP in LMICs, human RIG (HRIG) is expensive. In addition, the supplies of HRIG or RIG that is produced in animals such as horses (ERIG) are limited. Moreover, the risk of contamination from pathogens or unknown agents exists as HRIG and RIG are blood derived products [18].

Anti-rabies virus monoclonal antibodies (mAbs) can potentially overcome these limitations and serve as potent alternatives to RIG use in PEP. The first anti-rabies virus mAbs have recently gained regulatory approval in India [19, 20]. Several other candidates are under clinical evaluation. Given advances in the field, anti-rabies virus mAbs have been reviewed by the World Health Organization’s (WHO) Strategic Advisory Group of Experts (SAGE) on Immunization, and was included in the updated WHO rabies immunization policy recommendations [10]. Considering costs and supply limitations of HRIG and RIG, new WHO recommendations support more prudent use of RIG and encourage development of mAbs. In an updated position statement, WHO recommends that a registry be maintained to monitor the clinical use of mAbs and, as a research priority, supports development of biologics containing two or more mAbs with non-overlapping epitopes, to increase the efficacy and breadth of global rabies virus neutralization [21]. Considering these WHO recommendations, objective of this study was to develop mAbs that satisfy the following criteria: high virus neutralizing antibody (VNA) titers; an ability *in vitro* to cross react against diverse street viruses of public health relevance over representative continents (Africa, Asia, Europe, the Americas, etc.); efficacy *in vivo* against severe rabies virus challenge; and selection of different conformational epitope sites for broad complementarity to minimize the risk of viral mismatches under different field conditions and escape mutant evolution. The study conducted interference studies and ultimately selected two most potent mAbs from hundreds candidates, NP-19-9 and 11B6, which satisfied criteria set above.

## Materials and methods

Ethics statement: 1. Human blood: The experiment was approved by the Institutional Review Board (IRB) at the Seoul National University Hospital (IRB no H-1103-115-356). 2. Human blood: consent obtained written form. 3. Animal test at National Institute Mental Health and Neurosciences in India. 4. Animal test: The animal experiment was performed with prior approval by Institutional Animal Ethics Committee (IAEC) of National Institute Mental Health and Neurosciences in India (approval number: AEC/55/347/N.V) in accordance with national laws and policies.

### Separation of PBMCs from the blood of subjects vaccinated against rabies

The experiment was approved by the Institutional Review Board (IRB) at the Seoul National University Hospital (IRB no H-1103-115-356) and written consent form was prepared with the agreement of the volunteers. Healthy adult volunteers (n = 15) were vaccinated against rabies using commercial vaccines (Verorab^®^, Sanofi Pasteur). The volunteers were negative for anti-HCV and anti-HIV antibodies, and negative for other infectious viruses of major public health concern. Among volunteers, persons who has been vaccinated against rabies within a year were vaccinated once, and the others were vaccinated three times in total. One week after the final vaccination, approximately 50 mL of whole blood was collected. The peripheral blood mononuclear cells (PBMCs) were separated from the collected blood using the Ficoll-Paque PLUS method (Cytiva, Merck). The separated PBMCs were washed twice with phosphate buffered saline, adjusted to a concentration of 1x10^7^ cells/mL within a freezing medium, and stored in a liquid nitrogen tank. For the screening of antibodies against rabies virus G-protein, the PBMCs were subjected to phage display and B-cell sorting by FACS (fluorescence-activated cell sorting), respectively.

### Antibody screening by phage display

#### Phage library construction and biopanning

Phage display antibody library was constructed from the immunized human PBMCs as previously described [22]. Briefly, antibody variable regions (V_L_ and V_H_) were amplified by PCR with appropriate primers for phage display. cDNA, which was synthesized with total RNA extracted from the PBMCs, was used as template. Single-chain variable fragments (scFvs) were generated by linking V_L_ and V_H_ fragments and directly cloned into phagemid vector for library construction. After transformation of the scFv library into ER2738 cells and cultivation with helper phage, phages displaying scFv were harvested for biopanning to screen rabies virus G-protein-bound scFv displayed on phage. The rabies virus G-protein was coated on the well of the 96-well plate and incubated with the phage library. Following incubation, non-binders were washed out and only binding phages were eluted and infected into fresh ER2738 cells for two more rounds of biopanning. After three rounds of biopanning, several hundreds of clones were subjected to phage enzyme immunoassay and sequence analysis for further selection.

#### Phage enzyme immunoassay

Phage-infected ER2738 cells were plated, and individual colonies were inoculated for shake culture. When the OD600 reached 0.7 or higher, the VCSM13 helper phage was added to the cells, which were then cultured at 37°C for 12 hours or more. The culture was centrifuged to remove the host cells and to collect the supernatant containing phages. Phages were precipitated by adding 4% PEG and 0.5 M NaCl, and then centrifuged at 3000 g for 15 minutes. Precipitated phages were suspended with 1% BSA/TBS/0.02% NaN3. For a phage enzyme immunoassay, rabies virus G-protein was adsorbed onto a 96-well microtiter plate and blocked with 3% BSA/PBS. The phage supernatant prepared as described above was diluted with 6% BSA/PBS at a ratio of 1:1 and added to each well of the plate, followed by incubation at 37°C for 2 hours. Each well was washed three times with PBS containing 0.05% Tween 20, and then a horseradish peroxidase-labeled M13 antibody was added, followed by incubation at 37°C for 1 hour. Each well was washed three times with PBS containing 0.05% Tween 20 and then 2,2’-azino-bis [3-ethylbenzothiazoline-6-sulfonic acid]-diammonium salt (ABTs) was added. The candidate antibodies were selected by measuring absorbance at 405 nm.

### Antibody screening by B-cell sorting

#### Isolation of B-cells by FACS

Frozen PBMCs were thawed and B-cells were isolated using FACS to screen more candidate antibodies with different screening method. To increase the expression of antibodies in the thawed PBMCs, the PBMCs were cultured in a medium containing four cytokines (IL-4, IL-6, IL-21 and CD40L) at 37°C with 5% CO_2_ for 5.5 days. FACS was performed to isolate B-cells expressing rabies virus-specific antibodies from the cultured PBMCs. To obtain B-cells expressing rabies virus-specific antibodies, FITC-conjugated rabies virus G-protein was prepared, using an FITC conjugation kit (Abcam, Catalog No. ab102884). Labeled cells were isolated using FACS and plated onto a 96-well PCR plate with single cell.

#### Synthesis of cDNA in single cells and amplification of antibody genes

From separated single cells in each well of a 96-well plate, cDNA was synthesized using Superscript III First-Strand Synthesis System Kit (ThermoFisher Catalog No. 18080051). The synthesis of cDNA was performed according to the manual provided in the kit. An antibody gene was obtained from the synthesized cDNA using a modification of a method as previously described [23]. Briefly, two PCR steps were conducted. Firstly, V_H_ and V_L_ were amplified by PCR. In the second PCR step, the V_H_ and V_L_ were amplified again by nested PCR, using the primary PCR products as a template.

### Construction of full IgG DNA and preparation of full IgG antibody

In case of antibody screening by phage display, DNAs for V_H_ and V_L_ were synthesized following sequence analysis of the rabies G protein-bound scFvs. V_H_ and V_L_ DNAs were already obtained in the process of B-cell sorting. Those DNAs were cloned for conversion into full IgG DNAs. Specifically, V_H_ and V_κ_ DNAs were treated with *Not*I and *Age*I restriction enzymes and cloned into a modified pcDNA3.1 vectors containing constant region of IgG1 heavy chain or kappa light chain. V_λ_ DNA was treated with *Age*I and *Xho*I restriction enzymes and cloned into a modified pcDNA3.1 vector containing constant region of lambda light chain. Thereafter, heavy chain vector and light chain vector for each antibody were transiently expressed by co-transfection in Chinese hamster ovary (CHO) cells. Later, purification with affinity chromatography on Protein A (GE Healthcare, Catalog No, 28-9872-31 AA) was conducted to prepare intact IgG1 antibody.

### Antibody titer analysis by ELISA

A standard sandwich ELISA assay was used to assess the production levels of the secreted mAbs. Microtiter plates were coated with a goat anti-human IgG (γ-chain-specific) antibody (Merck, Catalog No. I3382). Plates were blocked with 1% bovine serum albumin in PBS. The captured product was detected using HRP-conjugated goat anti-human IgG κ-chain and substrate solution TMB. 1 N sulfuric acid was used to stop the reaction. The absorbance was measured at OD 450/650 nm using an ELISA plate reader. The titer of samples was calculated by comparison to a standard curve using a purified human IgG standard.

### Rabies virus G-protein purification

Baby hamster kidney (BHK) cells were infected with Evelyn-Rokitnicki-Abelseth (ERA) rabies virus strain and was incubated for four days the supernatant of virus-infected cells was harvested. Virus particles were collected using ultracentrifugation for 120 min at 50,000 × g, 4°C. The virus particle pellet was re-suspended in NTE buffer at pH 7.5. The virus was isolated on a 15–50% sucrose density gradient for 1 hour at 100,000 × g, 4°C. After centrifugation, the virus particles were obtained at a buoyant density of 1.17 g/cm^3^. Thereafter, 0.3 mol/L NaCl, 50 mmol/L trometamol-HCl (pH 7.6) and 2% OGP were added to the purified rabies virus. The mixture was incubated for 20 minutes at room temperature and centrifuged for 70 minutes at 120,000 × g. The solubilized rabies virus G-proteins were purified by isopycnic centrifugation on a sucrose gradient for 36 hours at 150,000 × g and 4°C. After centrifugation, the rabies virus G-protein was collected from the bottom of the tubes using a hypodermic needle.

### Standard Rapid Fluorescent Focus Inhibition Test (RFFIT)

The RFFIT was performed for detection of VNA, as previously described [24]. In brief, five-fold serial dilutions of mAbs were incubated with the CVS-11 rabies virus strain in 8-well tissue culture chamber slides for 90 minutes at 37°C. Mouse neuroblastoma (MNA) cells were added to the sample-virus mixture and incubated for an additional 20 to 24 hours at 37°C with 2 to 5% CO_2_. Slides were fixed in acetone and stained with a commercial anti-rabies (polyclonal) virus N-FITC-labelled conjugate (CUSABIO, Catalog No. CSB-PA321352LC01RAI). Twenty distinct microscopic fields per well were examined using a fluorescence microscope at a magnification of 160X to count the rabies virus-infected foci. The number of positive fields with rabies virus-infected foci per well was recorded. The neutralization endpoint titer was defined as the highest sample dilution at which 50% of the observed microscopic fields contained one or more rabies virus-infected foci. The RFFIT titers were interpolated using the Reed and Muench method. The endpoint VNA titer of the test serum was transformed into international units (IU)/mL by calibrating results against the endpoint neutralization titers of the Standard Rabies Immune Globulin reference (lot R-3, 59 IU) determined in the same assay run with an assigned potency value of 2.0 IU/mL.

### Modified fluorescent antibody virus neutralization (FAVN) test

An adaptation of the FAVN test was performed for detection of VNA [25]. In brief, three-fold serial dilutions of mAbs were incubated with 100 TCID_50_/well of diluted CVS-11 in 96-well tissue culture plates for 1 hour at 37°C with 5% CO_2_. Thereafter, BHK cells were added to the sample-virus mixture and incubated for 48–60 hours at 37°C with 5% CO_2_. Plates were fixed by adding 80% acetone and stained with anti-rabies virus mAbs (2C6 clone, ERA, Median Diagnostics, Catalog No. 9061), using an ABC kit (Avidin-biotin, Vector Laboratories, Catalog No. PK-4002) and a DAB peroxidase (horseradish peroxidase, HRP) substrate kit (Vector Laboratories, Catalog No.SK-4100). For detection of viral inclusions, we used ABC staining instead of fluorescence, and then an endpoint titer (i.e. the point at which 50% of the wells at that serum dilution showed the presence of rabies virus antigens) was calculated by the Spearman-Karber method. By convention, this titer was converted to a value in IU/mL by comparison to a standard reference serum with a known titer.

### Binding affinity of anti-rabies virus mAbs to rabies virus G-protein

An SPR analysis for binding affinity was performed using a CM5 sensor chip in a BIAcore T200 system at 25ºC. An HBS-EP buffer (GE Healthcare) was used as the running buffer during analysis. Purified rabies virus G-protein was immobilized on the sensor chip using the suggested amine coupling procedure. Each antibody (1 μM) was injected and bound to the immobilized rabies virus G-protein via a given association phase (120-s) at a constant flow rate of 30 μL/min. After a 100-second period of buffer flow, the secondary antibody (1 μM) was injected, followed by elimination of accumulated antibodies with the optimal regeneration buffers 10 mM glycine-HCl (pH 2.0) and 1 mM sodium hydroxide. At the end of each antibody association phase, the changed RU levels compared to each baseline were determined as a measure for binding.

### Epitope mapping

#### Shotgun mutagenesis epitope mapping with alanine scanning

For epitope mapping, shotgun mutagenesis with alanine scanning was conducted by Integral Molecular (PA, US). This technique use enabled the expression and analysis of large libraries of mutated target proteins with HEK-293 cells, which were transiently transfected and incubated overnight with either a rabies virus G-protein construct (rabies virus strain CVS-11, Genbank # AAC34683.1) or vector alone in a 384-well format, followed by immunodetection. Serial dilutions of each binding protein (beginning at 1 μg/ml) were tested for immunoreactivity against cells expressing wild-type rabies virus G-protein or vector alone. To avoid of avidity, this analysis was conducted in the form of Fab and every residue in a protein was individually mutated to an alanine and 20 customer-specified residues, to assay changes in its function.

### Generation of escape rabies virus variants and cDNA sequencing

An experiment to generate mAb-induced escape rabies virus variants was performed according to a previously described method [26]. Serial dilutions of CVS-11 rabies virus, ranging from 10^−1^ to 10^−7^ TCID50/mL were incubated with an appropriate amount (i.e., 1 and 4 IU/mL) of candidate mAbs for 1 hour at 37°C with 5% CO_2_. The mixtures were added to BHK-21 cells and then potential escape rabies virus variants were harvested three days after infection. The remaining cells on the plates were fixed and incubated with antibodies against rabies virus nucleoprotein, followed by staining with FITC-conjugated goat anti-mouse immune globulin G (Abcam, Catalog No. ab6785). Supernatants from wells showing one to four fluorescent foci were used to infect BHK-21 cells for virus amplification. The survival of the amplified virus was verified in the presence of 4 IU/mL of mAbs. The identified escape mutants were amplified, and total RNA was isolated using an RNeasy Mini Kit (QIAGEN, Catalog No. 74104). Subsequently, reverse transcription-PCR (RT-PCR) was performed using rabies virus-specific primers and a One-step SuperScript RT-PCR Kit with Taq DNA polymerase (ThermoFisher, Catalog No. 12574018). The cDNA was sequenced by standard procedures, as previously described [27].

### *In vivo* rabies virus challenge study

A mouse challenge study was performed using rabies virus isolated from infected dogs and humans in India. Field isolates were obtained from the brains by the mouse inoculation test as described by the WHO (SOP NIMH/NV/RAB 006). Isolates were stored as a mouse brain homogenate (20%) and titrated by inoculation into 4–6-week-old Swiss Albino mice. A total of 10 mice (average of 15–20 g) were used in each animal test group. The virus used consisted of 100 LD50 (in 0.1 mL) of each of the rabies viruses, by the intramuscular (gastrocnemius) route. After 3 hr of virus inoculation, each mAb was inoculated at the same site, after which the survival rate was observed for 30 days with Negative Control (PBS), Positive control (RIG), NP-19-9, 11B6 and mixtures at a dose of 20 IU/kg. The animal experiment was performed with prior approval by Institutional Animal Ethics Committee (IAEC) of National Institute Mental Health and Neurosciences in India (approval number: AEC/55/347/N.V) in accordance with national laws and policies.

### Membrane fusion inhibition assay

#### Rabies virus G-protein expressing cell line development

A DNA sequence for specific rabies virus G-proteins (ERA strain, GenBank #EF206707.1) was obtained from the National Center for Biotechnology Information (NCBI) database and cloned into a mammalian expression vector. This vector (MAR vector with pCT290, which is related to Celltrion’s patented MAR-based vector) was transfected to CHO-K1 cells (ATCC, CCL-61) using Lipofectamine^™^ LTX Reagent (ThermoFisher, Catalog No. 15338500), following the manufacturer’s instructions. The transfected cells were seeded and selected in each well of a 96-well plate using a SFM4CHO medium supplemented with 500 nM MTX. When the confluency of clones was over 70% of each well, the p-clones were scaled up sequentially to a 24-well plate, a 12-well plate, and a 6-well plate. Based on immunofluorescence assay results for the rabies virus G-protein of clones in the 6-well plate, clones producing G-protein at a high level were selected and scaled up to a 125-mL Erlenmeyer shake flask. After approximately 3 passages in a 125-mL Erlenmeyer shake flask, Research Cell Banks were prepared.

#### Fusion assay methods

For a membrane fusion inhibition assay, selected clones were cultured in a 6-well plate. Approximately 24 hours before this assay, rabies virus G protein-expressing CHO cells were seeded at 3x10^5^ cells/well into an assay medium (DMEM/F-12 medium containing 10% FBS). These cells were cultured in a humidified 5% CO_2_ incubator at 37°C. Antibodies were added (20 μg/mL) to the wells and incubated at 37°C for 1 hour. After washing with PBS, cells were exposed to pre-warmed, low pH buffer (150 mM NaCl, 10 mM HEPES, pH 5.0) and incubated at 37°C for 8 minutes. The acidic medium was replaced with DMEM supplemented with 10% FBS and cells were incubated for 1 hour to induce fusion activity. Cells were fixed with ice-cold methanol and then stained with trypan blue. Syncytia formation was evaluated by qualitatively observing random fields under an inverted microscope and photographed using a digital color CCD (Charge Couple Device) camera.

## Results

### Antibody screening with phage display and B-cell sorting

**Fig 1:**
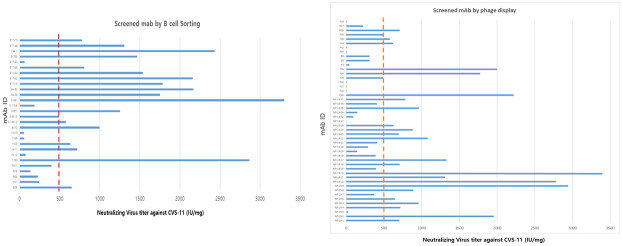
The VNA titers of anti-rabies mAbs. 75 mAbs were tested by VNA activity against CVS-11 and then 28 mAbs were selected with threshold of over 500IU/mg and titer.

To obtain human monoclonal antibody, PBMCs were collected from 15 vaccinated volunteers. Conventional phage display method was used to screen various anti-Rabies antibodies. However, heavy chain and light chain shuffling happens during the phage library construction therefore, single B cell selection was also used to screen more anti-rabies antibody with original heavy chain and light chain combination. several hundreds of phage clones were subjected to phage enzyme immunoassay and approximately 200 clones showed rabies G protein positive signal. Among them, 47 clones which showed highest signal were selected. A thousand of single B cells were sorted out and their heavy chain and light chain variable region genes were amplified by PCR from individual B cells, converted to full length immune globulin G (IgG) and then their binding to rabies G protein were confirmed. Through the B cell sorting, 28 clones which showed highest binding ability to rabies G protein were selected. In the first step, total 75 clones were selected from phage display and B-cell sorting method that were conducted in parallel and independently. To select the highly reactive mAbs against rabies virus, the VNA of 75 mAbs candidates were evaluated by FAVN or RFFIT using the CVS-11 strain (Fig 1). The neutralizing efficiency of each mAb was expressed as IU per mg. Among the 75 candidate mAbs, 28 candidates were selected based on their high potency (≥500 IU/mg VNA activity against CVS-11) and antibody titer in transient production.

### Selection of candidate mAbs by VNA ability against diverse viruses from six continents

To investigate the broad neutralizing activity of candidates, VNA assay was performed with several selected subtype of rabies virus. Since up to 99% of the cases of rabies infection in human are dog-mediated, one of the main strategies to develop anti-rabies mAbs firstly focused on neutralizing activity against canine rabies viruses. Therefore, 28 selected mAbs were tested with three representative canine rabies viruses (i.e., Gabon dog, India dog and China dog) in Asia and Africa [10, 21]. Besides canine isolates, VNA activity to other wildlife derived rabies viruses were also tested. This was in line with the previous study which confirmed the diversity and importance of wildlife rabies viruses [28]. As such, the study additionally tested VNA activity of mAbs to bat and skunk rabies viruses during screening. The results of the neutralizing test are shown in Table 1. In addition, this study group previously reported generation of several escape viruses and defined epitope groups of these escape viruses [28]. Likewise, for selected 28 mAbs, the study group not only considered VNA titer for selection but also epitope site, and finally selected 15 mAbs (NP-19-2, NP-19-5, NP-19-9, NP-19-10, NP-19-11, NP-19-13, NP-19-17, NP-19-35, 11B6, 3-9E1, Ba-36, Ba-53, B17-16, B17-50 and E30).

**Table 1:** The results of primary screening with six rabies viruses using RFFIT.

No	Virus	Antigenic Site	Dog, Gabon (IU/mg)	Dog, China (IU/mg)	Skunk, USA, California (IU/mg)	Bat, USA, Tennessee (IU/mg)	Dog, India (IU/mg)	Bat, USA, California (IU/mg)
	mAb							
1	NP-19-2	III	>1178	>329	>659	~1905	>2667	>3394
2	NP-19-5	I/IV	~842	>329	>659	0	>2667	424
3	NP-19-9	III	>1178	>329	>659	~2381	~2381	>3394
4	NP-19-10	III	>1178	>329	>659	~2381	>2667	>3394
5	NP-19-11	I/IV	>1178	>329	>659	~2143	~2571	~2182
6	NP-19-13	III	>1178	>329	>659	~2143	~2571	>3394
7	NP-19-17	I/IV	>1178	>329	>659	~2143	>2667	545
8	NP-19-22	III	~757	>329	>659	0	0	0
9	NP-19-24	III	>1178	>329	>659	0	~2381	424
10	NP-19-35	New	>1178	>329	>659	~2381	~2381	~3030
11	11B6	II	~2339	>3529	1438	~2381	3175	~10774
12	6A12	I/IV	3859	686	2157	Not tested	Not tested	Not tested
13	3-8F1	III	1781	~588	995	Not tested	Not tested	Not tested
14	3-9E1	III	>2947	>823	>1647	~4286	~6429	>3394
15	Ba-36	New	>1178	>329	>659	286	>2667	~2000
16	Ba-53	New	>1178	>329	>659	Not tested	>2667	~2424
17	B17-16	New	>1178	>329	Not tested	Not tested	>2667	2545
18	B17-42	New	~4962	>2353	0	0	1701	~10823
19	B17-50	New	>1178	>329	0	0	>2667	~3030
20	3-7B3	I/IV	~3828	>1467	>2995	~10823	~7153	0
21	E30	III	Not tested	Not tested	~1667	>2800	~2000	~2222
22	F29	III	Not tested	Not tested	>1867	0	>2489	489
23	F34	III	Not tested	Not tested	~833	>2800	~2222	~1778
24	F36	III	Not tested	Not tested	~1500	300	~2000	~2000
25	F44	Not tested	Not tested	Not tested	>1867	0	>2489	~622
26	F30	III	Not tested	Not tested	>1867	0	~2222	~578
27	F35	III	Not tested	Not tested	>1867	350	~2222	489
28	B29	Not tested	Not tested	Not tested	1400	0	~1867	~711

### Rabies VNA test of 15 selected mAbs against 47 worldwide Iyssaviruses

The VNA activity of 15 selected mAbs was tested against 47 lyssaviruses from around the world (S1 Table in S1 File). The objective of this test was to select mAb that has reactivity to broad-spectrum of lyssaviruses, along with potent virus neutralization activity. Table 2 shows the preliminary VNA activity of 15 mAbs against different viruses. While these values were not defined endpoints yet, they portrayed the relative ability of mAbs to neutralize each virus variants within a predictable range. In addition to the broad neutralizing activity of mAbs to various viruses, growth capacity of manufacturing cells, their suitability for commercial production (S1 Fig in S1 File) and epitope site of each mAb were evaluated for selection. As a result, of all 15 candidates, 3 mAbs were selected based on their neutralizing ability, antigenic sites, and complementarity as cocktails: NP-19-09, NP-19-35 and 11B6.

**Table 2:** The VNA activity test for 15 mAbs against selected Iyssaviruses.

No.	Virus	Antibody Clone Name and Antigenic Site														
		Antigenic Site 1 or 4		Antigenic Site 2	Antigenic Site 3					Unknown or Undetermined Antigenic Site						
		NP-19-5	NP-19-17	11B6	NP-19-2	NP-19-9	NP-19-10	NP-19-13	E30	NP-19-11	NP-19-35	3-9E1	Ba-36	Ba-53	B17-16	B17-50
1	CVS-11	>900^a^	>1300	~2800 ^b^	>1900	>2900	>2700	>4800	>2000	>1300	>960	>5600	>1750	>2100	>1780	>1530
2	Mongoose, South Africa	>1018	>1018	>1018	>1018	>1018	>1018	>1018	>1018	>1018	>1018	>1018	>1018	>1018	>1018	>2036
3	Skunk, California, USA	≥2000	≥2000	≥2000	≥1929	>2000	>2000	>2000	>2000	>1929	>2000	~1786	>2000	>2000	0	0
4	Dog, Tunisia	>2000	>2000	>2000	>2000	>2000	>2000	>2000	>2000	>2000	>2000	~1786	>2000	>2000	>2000	>2000
5	Dog, Gabon	~4762	~4000	~2333	>5333	>5143	>5333	>5333	>5333	>5143	>5143	~4000	>5333	>5333	>5333	>10667
6	Gray fox, Texas, USA	>1318	>1318	>847	>1318	>1176	>1318	>1318	>1318	>1271	>1318	>1318	>1318	>1318	>1318	>1318
7	Dog, Thailand	~365	~435	>487	>487	>487	>487	>487	>487	>487	>487	>470	>487	>487	>487	~939
8	Dog, Mexico	>896	>896	>896	>896	>896	>896	>800	>896	>896	>896	>896	>896	>896	>896	>896
9	Human/dog, Philippines	>400	>400	>400	>400	>400	>400	>400	>400	>400	>400	>400	>400	>400	>400	Not tested
10	Bat, Mexico	>896	>896	>896	>896	>896	>896	>896	>896	>896	>896	>896	>896	>896	>896	>800
11	Bat, Brazil	>400	>400	>400	>386	>400	>400	>386	>400	>400	>386	>400	>400	>400	>400	~643
12	Human/dog, Philippines	>400	>400	>400	>400	>400	>400	>400	>400	>400	>400	>400	>400	>400	~286	~643
13	Bat, Washington, USA	>448	>448	>448	>448	>448	>448	>448	>448	>448	>448	>448	>448	>448	>448	Not tested
14	Dog, Argentina	>400	>400	>400	>400	>400	>400	>400	>400	>400	>400	>400	>400	>400	0	0
15	Skunk, Texas, USA	~1493	>1493	~1493	>1493	>1493	>1493	>1493	>1493	>1493	>1493	>1493	>1493	>1493	>1493	~1333
16	Raccoon, Georgia, USA	>2667	~1381	>1190	~1905	>2571	>2667	905	~1905	1190	667	333	>2667	>2667	>2667	1810
17	Dog, China	>448	>448	>448	>448	>448	>448	>448	>448	>448	>448	>448	>448	>448	>448	>896
18	Cow/dog, China	>400	>400	>400	>400	>400	>400	>400	>400	>400	>400	>400	>400	>400	>400	>400
19	Coyote, Texas, USA	>896	>896	>640	>896	>896	>896	>896	>896	>896	>896	~800	>896	>896	>896	~1728
20	Human/dog, UK	~400	>448	>448	>448	>448	>432	>448	>448	>448	>448	>400	~400	>448	>448	>432
21	Bat, Alabama, USA	>2000	>2000	>1786	>2000	>2000	>2000	>2000	~1786	>2000	>2000	>2000	>2000	>2000	>2000	>4000
22	Bat, New York, USA	0	~714	>700	~1786	>1786	~1786	>1600	~1143	~1429	~714	~943	~1200	~829	200	0
23	Bat, Pennsylvania, USA	>255	~246	>1500	~227	>255	>255	~227	~246	~227	>255	>255	>255	>255	~227	100
24	Bat, California, USA	>1333	>1493	>1493	>1493	>1493	>1493	>1493	>1493	>1493	>1493	~1333	>1493	>1493	>1493	>2987
25	Bat, Arizona, USA	N/A	>2240	~1240	>2240	>2240	>2240	~1786	>2240	~520	>2240	~520	>2240	~1000	0	0
26	Bat, Virginia, USA	N/A	440	~1320	280	>2000	~2000	~2160	>2240	~2000	~2000	0	0	0	0	0
27	Bat, Tennessee, USA	N/A	429	>2381	~286	>2000	>2667	>2667	~2143	>2667	~2381	0	0	0	0	0
28	Bat, Tennessee, USA	N/A	546	~3394	~3030	>3030	>3394	~3030	~3030	>3394	>3394	364	~1152	~1030	0	0
29	Skunk, Texas, USA	>400	>400	>400	>400	>400	>400	>400	>400	>400	>400	>400	>400	>400	>400	>800
30	Arctic fox, Alaska, USA	>448	>448	>448	>448	>448	>448	>448	>448	>448	>448	>448	>448	>448	>448	>896
31	Raccoon dog, Russia	>207	>207	>207	>207	>207	>207	>207	>207	>207	>207	>207	>207	>207	>207	Not tested
32	Dog, India	>448	>448	>448	>448	>448	>448	~360	>448	>448	>448	>448	>448	>448	>448	>896
33	Mongoose, Puerto Rico	~500	>560	>560	>560	>560	>560	~500	>560	>560	>560	>560	>560	>560	~500	~1000
34	Gray fox, Arizona, USA	>2074	>2074	>2074	>2074	>2000	>2074	>2074	>2074	~1852	>2074	370	>2074	>2074	>2074	519
35	Skunk, Wisconsin, USA	>400	>400	>400	>400	>400	>400	>400	>400	>400	>400	>400	>400	>400	>400	>800
36	Dog/coyote, Texas, USA	>400	>448	>448	>448	>448	>448	>448	>448	>448	>448	>448	>448	>448	0	0A
37	Bat, Tennessee, USA	0	407	~2000	~1074	~1852	~2000	>2074	>2000	>2000	~1556	0	0	0	0	0
38	Dog, India	~1905	~1571	>2571	>2571	>2571	>2667	~1905	>2381	>2381	>2381	~809	~2143	~1905	~1714	~1333
39	Bat, Tennessee, USA	0	440	~2240	>2240	~2240	>2240	>2240	>2240	>2240	>2240	0	0	~560	0	0
40	Cow, Sri Lanka	>772	>772	>772	>772	>772	>772	>772	>772	>772	>772	>772	>772	>772	>772	>1545
41	Bat, Washington, USA	>448	>448	>448	>448	>448	>448	>448	>448	>448	>448	>448	>448	>448	>448	>896
42	Australian bat lyssavirus	>448	>448	>448	>448	>448	>448	>448	>448	>448	>448	>448	>448	>448	>448	>896
43	ABV (SM 4476), Australian bat lyssavirus	>896	>896	>896	>896	>896	>896	>896	>896	>896	>896	>896	>896	>896	>896	>896
44	ERA	>432	>448	>448	>448	>448	>448	>448	>448	>448	>448	>448	>448	>448	>448	>896
45	EBLV 1 lyssavirus, A09-3484	0	0	>8615	>8615	>8615	>8615	>8615	>8615	>8307	>8615	~3231	~2923	~2615	>8615	~4308
46	EBLV 2 lyssavirus, A03-4659	0	0	>7200	~10800	>11200	>11200	>11200	>11200	>11200	>11200	0	~6600	0	>11200	4400
47	Duvenhage lyssavirus	0	0	>8615	>8615	>8615	>8615	>8615	>8615	>8615	>8615	~3846	0	0	>8615	3385

Neutralizing potency against viral field isolates was measured in a standard RFFIT and indicated as follows:

all results are in IU/mg unit and neutralizing potency of mAbs is normalized by the neutralizing potency of HRIG.

> sign means that the accurate neutralizing potency of candidate mAbs could not be determined because these mAbs neutralized the viruses at the highest dilution factor in the tested range.

~ sign means that the expected neutralizing potency of the candidate mAbs was at the maximum level.

### Determination of the epitope of candidate mAbs

To determine the exact epitope site of the selected mAbs, escape mutant test was conducted [26]. The amino acid variants that were identified as key binding epitopes of NP-19-9 in the escape mutant studies were detected on amino acid 331 which is belong to an antigenic site III. The 11B6 mAb had loss of neutralizing activity against 34 amino acid variant of escape viruses and determined an antigenic site class II. However, NP-19-35 has a non-neutralization strain of escape mutant in amino acid on 201 and 413 (Table 3), which is not related to known antigenic sites. Having novel antigen binding site, NP-19-35 was considered to be of more academic value than industrial purposes. Though NP-19-35 showed high VNA activity, novel epitope of NP-19-35 would render it difficult from progression to clinical trials and being approved as new therapeutic agent. Therefore, it was eliminated from mAb candidate, yet would be put in for other scientific studies for academic purposes.

**Table 3:** Determination of antigenic sites for the mAbs.

Antibody	Antigenic Site	Codon Change	Amino Acid Change	Amino Acid Position *
NP-19-9	III	TCA→ CCA	Ser(S)→ Pro(P)	331
NP-19-35	Unknown	GTG→ GAG	Val(V)→ Glu(E)	210
		GAG→ GAT	Glu(E)→ Asp(D)	413
11B6	II	GGA→ GTA	Gly(G)→ Val(V)	34
		GGA→ GAA	Gly(G)→ Glu(E)	34
		GGA→AGA	Gly(G)→ Arg(R)	34

* Amino acid position refers to the position number (except for the signal peptide) of the rabies virus G-protein.

#### Confirmation of epitope site of candidate antibody via alanine scanning

**Fig 2:**
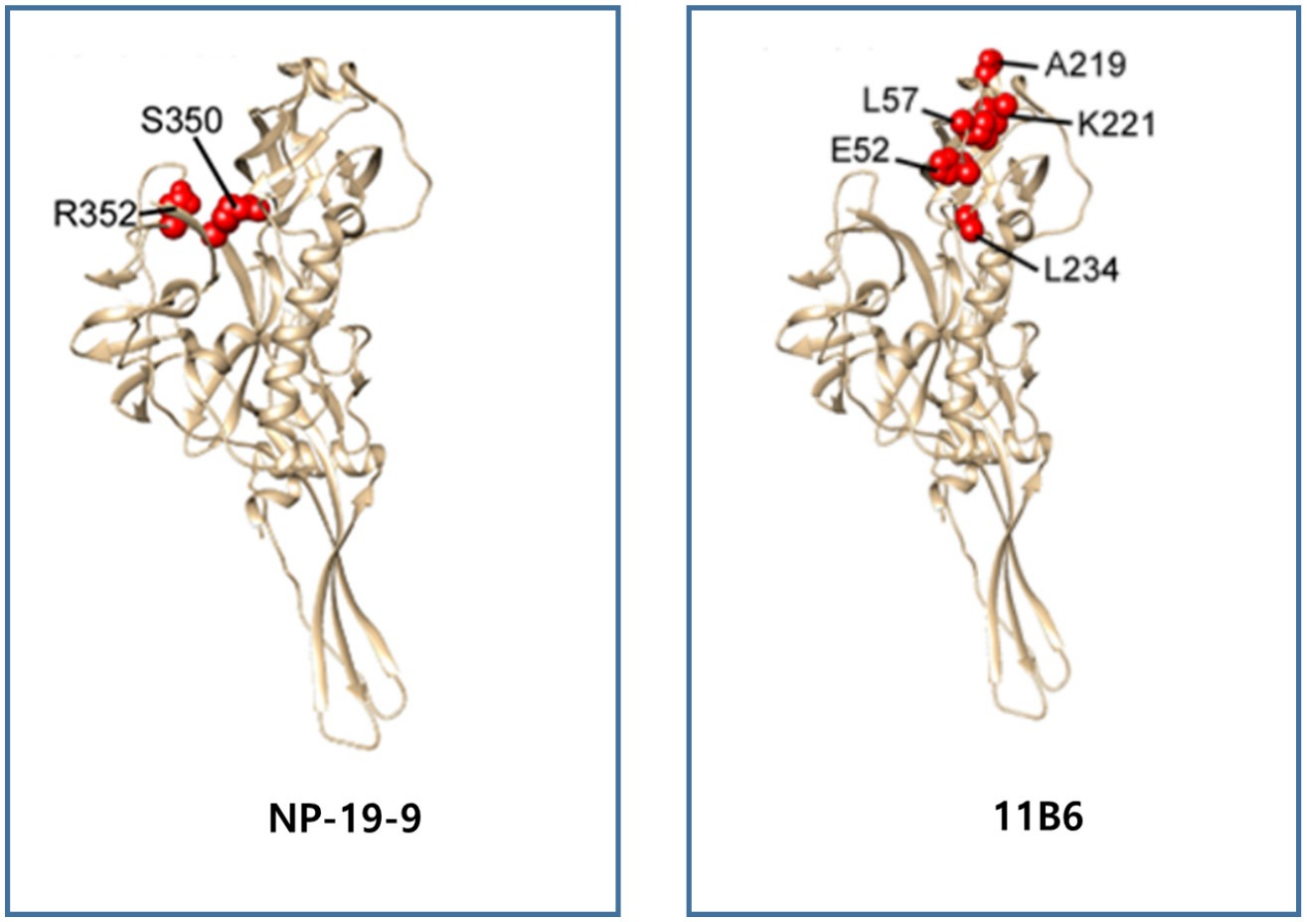
Identification and visualization of critical residues for NP-19-9 and 11B6. Since there was no crystal structure of rabies viruses available to approximate the epitope locations, the residues are highlighted on the crystal structure of prefusion vesicular stomatitis virus (VSV) glycoprotein G [29], which has approximately 20% identity with rabies viruses. Critical residues that have no homologues on the VSV-G crystal structure (G53 and C54) were not plotted.

To clarify the epitope of final mAb candidates, NP-19-9 and 11B6, the binding affinity was tested using alanine scanning of the mutation library. Through the screening of the rabies virus mutation library with the NP-19-9 and 11B6, critical residues representing amino acids whose side chains make the highest energetic contributions to the mAb-epitope interaction were validated [29]. Screening with the NP-19-9 identified one critical residue, R352. Screening specific mutations also identified an additional residue, S350, which may also be part of the NP-19-9 epitope. Both R352 and S350 are in the previously characterized rabies virus antigenic site III [29]. Screening with the 11B6 identified seven critical residues for binding—E52, G53, C54, L57, A219, K221 and L234. Four of these residues (G53, C54, L57 and A219) were in the previously characterized rabies virus antigenic site II [30]. Through the escape mutant test and alanine scanning, we detected the final epitope site of NP-19-9 and 11B6 (Fig 2 & Table 4).

**Table 4:** Alanine scanning for mAbs 11B6 and NP-19-9.

mAb ID	Amino Acid Residue			Antigenic Site
	No.	Change of Amino acid	Amino Acid Site*	
NP-19-09	1	Ser(S)-->Leu(L)	331 (350)	Antigenic site III
	2	Arg(R)-->Ala(A)	333 (352)	Antigenic site III
11B6	1	Gly(G)-->Ala(A)	34 (53)	Antigenic site II
	2	Cys(C)-->Ala(A)	35 (54)	Antigenic site II
	3	Leu(L)-->Ala(A)	38 (57)	Antigenic site II
	4	Ala(A)-->Ser(S)	200 (219)	Antigenic site II

*Amino acid site excluding the signal peptide of rabies virus G-protein.

### Examination of neutralizing abilities of NP-19-9 and 11B6

Previously, the preliminary neutralizing activity was tested for 15 mAbs against 47 lyssaviruses found globally. For focal characterization of neutralizing activity of NP-19-9 and 11B6 to previously tested viruses, *in vitro* RFFIT test were conducted with an endpoint study. The results of the test are shown in Table 5. Overall, NP-19-9 antibody showed high neutralization activity across tested viruses. 11B6 showed slightly higher neutralization against bat viruses in Mexico, Brazil and the USA (i.e., Washington State). Rabies-related lyssaviruses from Africa, Australia and Europe were also neutralized by both mAbs.

**Table 5:** Neutralization data using the RFFIT for human mAbs against diverse lyssaviruses.

No.	Virus	NP-19-9 (IU/mg)	11B6 (IU/mg)
1	Rabid Cow (CVS-11)	7353	2059
2	Mongoose, South Africa	9143	7714
3	Skunk, California, USA	2160	2000
4	Dog, Tunisia	2700	2300
5	Dog, Gabon, Africa	5313	2031
6	Gray fox, Texas, USA	9259	2074
7	Dog, Thailand	9286	2286
8	Dog, Mexico	8800	2720
9	Human/dog, Philippines	2500	2500
10	Bat, Mexico	1714	2381
11	Bat, Brazil	1800	2240
12	Dog, Philippines	5185	1389
13	Bat, Washington, USA	2000	2160
14	Dog, Argentina	2125	1813
15	Skunk, Texas, USA	3571	1071
16	Raccoon, Southeast USA	5370	3519
17	Dog, China	2321	2143
18	Cow/dog, China	11818	2545
19	Coyote, Texas, USA	5000	1647
20	Human/dog, United Kingdom	2435	1739
21	Bat, Alabama, USA	4800	2533
22	Bat, New York, USA	10714	2619
23	Bat, Pennsylvania, USA	5263	5263
24	Bat, California, USA	7586	7586
25	Bat, Arizona, USA	11304	1087
26	Bat, Virginia, USA	13889	2778
27	Bat, Tennessee, USA	15600	5800
28	Bat, Tennessee, USA	26667	2533
29	Skunk, Texas, USA	2800	2000
30	Fox, Alaska, USA	10000	2222
31	Raccoon dog, Russian Far East	2700	2800
32	Dog, India	9412	3676
33	Mongoose, Puerto Rico	2000	1929
34	Gray fox, Arizona, USA	28333	2667
35	Skunk, Wisconsin, USA	2160	2240
36	Dog/coyote, Texas, USA	17361	1667
37	Human/wolf, Russia	1556	2000
38	Bat, Tennessee, USA	50000	10000
39	Dog, India	2000	2600
40	Bat, Tennessee, USA	12500	1964
41	Cow, Sri Lanka	2000	2160
42	Bat, Washington, USA	10000	8214
43	Bat, Australia (Wu ABLV)	3385	1923
44	Bat, Australia (ABV (SM 4476))	7778	2000
45	ERA	8929	1786
46	EBLV 1 A09-3484	27000	2800
47	EBLV 2 A03-4659	15556	3000
48	Duvenhage	36000	2200
49	EBLV 1 A09-3485	33333	9259
50	EBLV 2 A09-3483	27778	11111

### Binding affinity test with Surface Plasmon Resonance (SPR)

A SPR assay was used to evaluate the binding affinity of NP-19-9 and 11B6 to rabies virus antigen through measurement of the forward and reverse reaction rate constants. As shown in Table 6, NP-19-9 and 11B6 both have a high binding affinity to the rabies virus G-protein.

**Table 6:** Binding affinity of NP-19-9 and 11B6 to rabies virus G-protein.

Sample	ka1 (1/Ms)	kd1 (1/s)	KD	AVR KD
NP-19-9	2.11Ⅹ106	5.74Ⅹ10–4	2.72Ⅹ10–10	2.24Ⅹ10–10
	1.54Ⅹ106	2.70Ⅹ10–4	1.75Ⅹ10–10	
11B6	2.52Ⅹ105	9.39Ⅹ10–4	3.73Ⅹ10–9	4.54Ⅹ10–9
	2.14Ⅹ105	1.15Ⅹ10–3	5.36Ⅹ10–9	

### Evaluation of the interference of NP-19-9 and 11B6 as a cocktail

#### Interference test of the mAb cocktail with bioassay, *in vitro*

To investigate whether the mAbs could cause an interference with each other when administered together as cocktail against rabies virus, RFFIT test was performed using NP-19-9, 11B6 and a mixture of two (1:1 ratio, considering the potency level) against 13 rabies viruses, from different continents and hosts. Based upon comparative neutralizing activity, it was confirmed that there is no interference on biological activity of each mAb when administered together (S2 Table in S1 File).

#### Interference test with molar excess

Given that two mAb candidates are aimed to be administered together as cocktail as recommended by the WHO, competition may arise between the two mAbs which may result in change in efficacy. To test whether co-administration of NP-19-9 and 11B6 results in competition that affect and efficacy, cocktail reagents with different concentration of ratio of NP-19-9 and 11B6 were prepared. This was to determine if interference can arise depending on the each mAb concentration. The RFFIT was performed three times per sample of various concentration ratio of the two mAbs from 1: 0.1 and 1: 9. As shown in Table 7, the result demonstrated no significantly different levels were found under all different concentration ratio of mAbs tested. Additionally, Dunnnett’s test was performed for statistical analysis. Briefly, Dunnett’s test retrieves expected p-value of the ${\overset{-}{X}}_{i}-{\overset{-}{X}}_{control}$ difference, evaluating whether differences between the mean from the treatment group and the control group is significant. If the p-value is lower than α (significant level: 0.05), one can conclude that there is a significant difference between the two groups and their interaction, and vice versa. The value was calculated using SAS 9.4 (Cary, NC, USA, 64-bit).

**Table 7:** Interference test with CVS-11 rabies virus strain by the RFFIT.

**(a) Based on the concentration of 11B6**							
RFFIT data (IU/mg), measurement based on the concentration of 11B6							
Group	11B6 (%)	NP19-9 (%)	1	2	3	Mean	SD
	0	100	N/A	N/A	N/A	N/A	N/A
A	10	90	45000	34185	102556	60581	36752
B	25	75	41023	18000	23688	27570	11992
C	50	50	6837	3948	11844	7543	3995
D	75	25	2000	1519	3464	2328	1013
E	90	10	962	731	1667	1120	487
Control	100	0	380	380	658	473	161
**(b) Based on concentration of NP-19-9**							
RFFIT data (IU/mg), measurement of based on concentration of NP-19-9							
Group	11B6 (%)	NP19-9 (%)	1	2	3	Mean	SD
Control	0	100	7793	4500	10256	7516	2888
A	10	90	5000	3798	11395	6731	4084
B	25	75	13674	6000	7896	9190	3997
C	50	50	6837	3948	11844	7543	3995
D	75	25	6000	4558	10391	6983	3038
E	90	10	8659	6580	15000	10080	4386
	100	0	N/A	N/A	N/A	N/A	N/A

The statistical results in Table 8 show that most of the calculated p-values for the mean difference between the treatment groups and the control group were higher than α stating that there is no significant interference effects between the comparators. However, one statistically significant interference between 10% 11B6 and 90% NP19-9 was shown as seen in panel B of Table 8. Since the mAb NP-19-9 renders atrong neutralizing activity when NP-19-9 comprised 90% of the mAbs mixture, the calculated P value demonstrated a significantly different result. However, neither interference nor growing trend of interference was shown from increasing either of the antibody proportion to the other. Therefore, though one result showed statistically significant interference from molar excess of NP-19-9 over 11B6, it was concluded that there is no significant interference between the two mAbs, when administered together.

**Table 8:** Multiple neutralization test comparisons of the mAbs (RFFIT against CVS-11 rabies virus strain).

**(a) Based on concentration of 11B6**							
Minimum square approximation							
Multiple comparisons	Estimated value	SD	Degrees of freedom	t-value	Probability > |t|	Modified p-value	Result
A vs Control	-785.08	3080.8	12	-0.25	0.8032	0.9991	Not significant
B vs Control	1673.77	3080.8	12	0.54	0.5969	0.9723	Not significant
C vs Control	26.7002	3080.8	12	0.01	0.9932	1	Not significant
D vs Control	-533.29	3080.8	12	-0.17	0.8655	0.9999	Not significant
E vs Control	2563.39	3080.8	12	0.83	0.4216	0.8691	Not significant
**(b) Based on concentration of NP-19-9**							
Minimum square approximation							
Multiple comparisons	Estimated value	SD	Degrees of freedom	t-value	Probability > |t|	Modified p-value	Result
A vs Control	60108	12961	12	4.64	0.0006	0.0024	significant
B vs Control	27098	12961	12	2.09	0.0585	0.1958	Not significant
C vs Control	7070.4	12961	12	0.55	0.5954	0.9718	Not significant
D vs Control	1855.1	12961	12	0.14	0.8886	0.9999	Not significant
E vs Control	647.41	12961	12	0.05	0.961	1	Not significant

### Efficacy of NP-19-9, 11B6, and cocktail administration to Indian rabies virus *in vitro* and *in vivo*

**Fig 3:**
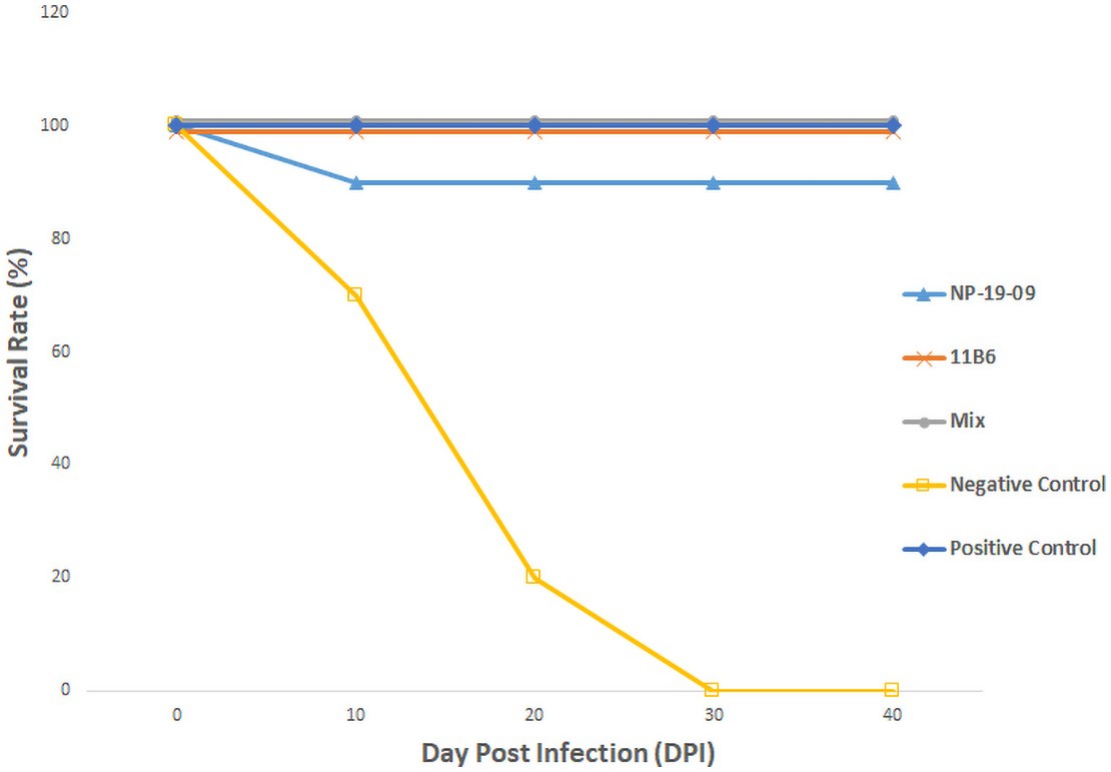
Efficacy of mAbs after rabies virus infection. Ten mice were tested and were injected with 100MICLD50 rabies virus before mAb administration. The graph demonstrates either NP-19-9 or 11B6 alone and cocktail administration all effectively protect tested mice from rabies virus infection.

The neutralizing activity of individual NP-19-9 and 11B6, cocktail (NP-19-9 and 11B6) against six rabies viruses that were isolated in India were tested using the RFFIT and an animal viability test. Each test group was prepared at a concentration of approximately 1 μg/ml and was carried out in Neuro-2a cells with 100 FFD_50_ of each virus (S3 Table in S1 File). The RFFIT results showed a neutralizing activity against each six virus by all mAbs group tested (S4 Table in S1 File). For animal viability test, mice were infected with rabies virus SV1-SV6 (S4 Table in S1 File) and was treated either negative control, HRIG, NP-19-9, 11B6, or cocktail (NP-19-9 and 11B6). Representative survival rate change from SV2 infection and treatment is shown in Fig 3. One month following testing, all mice treated with HRIG, NP-19-9, 11B6, or a mAb cocktail (NP-19-9 and 11B6) remained over 90% of survival rate, whereas mice treated with PBS, negative control, developed signs of rabies, varying from 7 to 20 days post infection. The result demonstrated that NP-19-9 and 11B6, and their cocktail effectively neutralized the Indian rabies virus isolates.

### Membrane fusion inhibition assay

**Fig 4:**
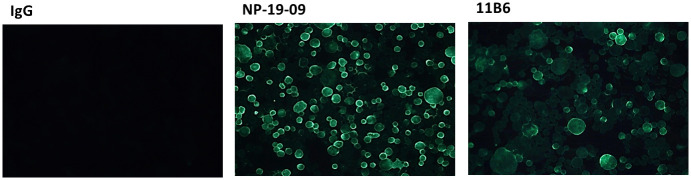
Binding affinity test between mAbs and ERA G protein expression cells. NP-19-9 and 11B6 have binding capacity to the ERA G protein expression cells.

To clarify whether NP-19-9 and 11B6 mAbs can inhibit cellular membrane fusion by interfering with the low-pH-induced conformational change, membrane fusion inhibition assay was conducted. Using the ERA (ABN93191.1) G protein expression cells, we confirmed binding affinity (Fig 4) of NP-19-9 and 11B6 antibody to the rabies expressing cells. As shown in Fig 4, only NP-19-9 and 11B6 binds to expression cells giving fluorescence, whereas non-specific IgG (i.e., the negative control) did not.

**Fig 5:**
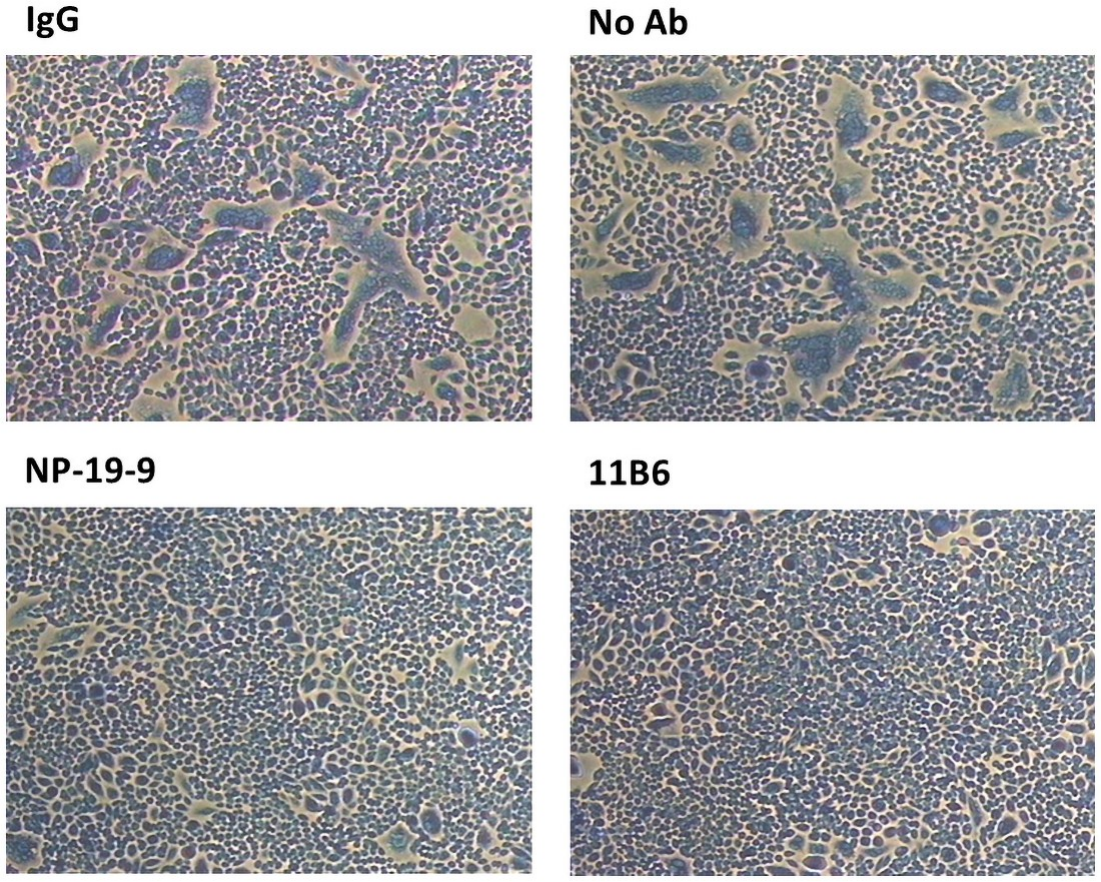
Inhibitory effect of anti-rabies virus mAbs on ERA G-protein-induced membrane fusion assay. Cells were treated with TPCK-trypsin and were subject to low pH condition. Syncytia formation is apparent for the control group (up, IgG and Control) whereas when treated with NP-19-9 or 11B6, syncytia formation is drastically decreased.

The CHO cells expressing specific rabies virus G-proteins were treated with either TPCK-Trypsin and NP-19-9, 11B6 or a non-relevant control antibody. The CHO cells treated with the control antibody were fused and formed syncytia during this process, as shown in Fig 5. However, both NP-19-9 and 11B6 inhibited syncytia formation of CHO cells expressing the ERA rabies virus strain G-protein (Fig 5). The low pH transition involves irreversible conformational changes that help deliver viral peptide to the target cell, inhibiting cell fusion in low-pH condition is critical in suppressing viral activity. Thus, the fusion inhibition activity of NP-19-9 and 11B6 correlated with their ability to neutralize cell-adsorbed rabies virus.

## Discussion

Procuring effective and affordable RIG in LMICs is a vital issue. Though rabies infection can be treated and further disease progression can be prevented via PEP with RIG infiltration, it is reported that some region in Asia and Africa still has limited access to RIG [31]. Strikingly, it is also reported that even category III case were unavailable in many countries [32]. As such, mAbs offer a promising alternative to RIG in resolving limited supply and high cost, especially for HRIG. Developing anti-rabies mAbs could also help abating possible safety concern from applying RIG, blood-derived products which is never safe from contamination. Also, mAbs production is free from animal welfare concern, as does in case or ERIG.

As mAb development can address public health concern for LMICs, this study aimed to develop potent mAb to be used for PEP. Considering previous studies and WHO recommendations for the development of broad-spectrum anti-rabies virus mAbs, the study tested the neutralizing activity of human mAbs against multiple lyssaviruses isolated from different hosts and countries. The study has also taken epitope site of the mAb and cocktail use of mAbs into account. Altogether, two potent mAbs, NP-19-9 and 11B6 was selected for their ability to neutralize a broad spectrum of global lyssaviruses. The two antibody was selected as each recognized different antigenic sites (II and III). As both mAb showed no interference in rabies virus neutralizing activity when used together, these mAbs was considered optimal candidate to be used as a cocktail mAbs therapeutic reagent for rabies prophylaxis. All rabies virus used for the study were especially from the phylogroup 1 closely related to most human rabies infection case (S5 Table in S1 File) [33, 34].

In an early screening stage, hundreds of candidates were produced. The mAbs were screened considering several properties, including viral neutralization titer, antibody production capacity, production cell line stability and epitope site. In the initial neutralization tests, the dilution factor was adjusted to verify the capability of antibodies within a predictable range. After when final two mAbs were selected, the exact dilution drainage or factor was applied to determine the neutralization capacity level of the antibody (Table 5, endpoint study). Since the study selected and developed mAbs that overcome the limitation of a single product, the two antibody—NP-19-9 and 11B6 –were considered for cocktail us. Each antibody had a different antigenic site, as this was already considered from the beginning of the screening.

In addition, several escape mutant viruses from previous study were obtained to distinguish the epitope of candidate mAbs. Once two mAbs were identified using the escape testing, additional confirmation was conducted by alanine scanning. Thereafter, the two mAbs were recertified through a bio-assay and a molar excess test. In the bio-assay case, neutralizing activity of a mAb cocktail against 12 rabies virus variants were tested by RFFIT. Because the concentration of the mAb cocktail was adjusted to a 1:1 ratio, a change in neutralization was observed. However, no significant reduction in neutralization in the competition effect was observed. The molar excess test checked whether there was an interference or competitive effect between the two mAbs when used at different concentrations. No interference or competitive effect was found, based on the neutralization capability value of each concentration.

Epitope site of several candidate mAbs, including NP-19-35, was not defined among known antigen binding sites [28, 29]. In an escape mutant test, amino acid variants that were identified as key binding epitopes of NP-19-35 were turned out 210 and 413 amino acid sequence in rabies G protein. It means that the epitope site of NP-19-35 was not belong to known antigenic site.

Efficacy of the selected mAbs was confirmed via studies in mice, against rabies viruses isolated in India (Fig 3). These street viruses were isolated from different regions in India and applied to the experiment. Administration of either NP-19-9 or 11B6 mAb after rabies virus infection mice resulted in a survival rate of over 90%. Finally, 100% survival rate was shown when two mAbs were co-administered as cocktail.

To define the potential mechanism of action of mAbs, we performed a fusion assay using a specifically designed cell line. In previous studies, we studied the mechanism of action in which antibodies act on a particular virus, focused upon how viruses are blocked by antibodies after endocytosis [35, 36]. In this study, a membrane fusion inhibition assay confirmed that low-pH-induced conformational change and cell-cell fusion were inhibited by NP-19-9 and 11B6. These mAbs inhibited viral genome release into the cytosol by blocking membrane fusion between the viral envelope and the endosomal membrane.

In conclusion, we identified two potent mAbs, NP-19-9 and 11B6 to be taken for further clinical research for clinical application. These mAbs have high potency, bind to different antigenic sites (II and III), have no competitive or interference effects, cross-protect against escape variants and neutralize a broad range of lyssavirus isolates. In addition, efficacy studies demonstrated significant protection induced from NP-19-9 and 11B6 cocktail administration when tested against a lethal rabies virus challenge compared to controls. These results suggest that the selected mAbs are strong candidate to be clinically used for PEP and rabies control within LMICs.

## Supporting information

S1 File (DOCX)
