# Supplementary material for: A broad-spectrum and highly potent human monoclonal antibody cocktail for rabies prophylaxis
Source: PLoS One. 2021 Sep 1;16(9):e0256779. doi: 10.1371/journal.pone.0256779 (PMC8409651; doi:10.1371/journal.pone.0256779)
Supplement: S1 File — (DOCX) [file pone.0256779.s001.docx]

**S1 Table. The list of rabies viruses from the U.S. CDC**

| **No.** | **Lyssavirus (Isolation source, country)** | **Identification number*  or  Date of isolation** |
| --- | --- | --- |
| 1 | Rabid cow, France (CVS-11) | 1882^&^ |
| 2 | Mongoose, South Africa | A07-0434 |
| 3 | Skunk, California, USA | A12-1826 |
| 4 | Dog, Tunisia | A07-0433 |
| 5 | Dog, Gabon, Africa | A04-2030 |
| 6 | Gray fox, Texas, USA | A04-0717 |
| 7 | Dog, Thailand | A04-2031 |
| 8 | Dog, Mexico | A04-2029 |
| 9 | Human/dog, Philippines | A07-0448 |
| 10 | Bat, Mexico | NA^%^ |
| 11 | Bat, Brazil | A04-2032 |
| 12 | Dog, Philippines | A07-0447 |
| 13 | Bat, Washington, USA | A04-0723 |
| 14 | Dog, Argentina | A04-2028 |
| 15 | Skunk, Texas, USA | A04-0714 |
| 16 | Raccoon, Southeast USA | A04-0712 |
| 17 | Dog, China | A07-0445 |
| 18 | Cow/dog, China | A07-0446 |
| 19 | Coyote, Texas, USA | A04-0718 |
| 20 | Human/dog, United Kingdom | A07-0439 |
| 21 | Bat, Alabama, USA | A04-0720 |
| 22 | Bat, New York, USA | SM: 44, date of sample collection 14 Jan 2004 |
| 23 | Bat, Pennsylvania, USA | A04-2024 |
| 24 | Bat, California, USA | A07-0449 |
| 25 | Bat, Arizona, USA | A04-0721 |
| 26 | Bat, Virginia, USA | A07-0454 |
| 27 | Bat, Tennessee, USA | A07-0456 |
| 28 | Bat, Tennessee, USA | A07-0450 |
| 29 | Skunk, Texas, USA | A07-0457 |
| 30 | Arctic fox, Alaska, USA | A04-0711 |
| 31 | Raccoon dog, Russian Far East | A07-0436 |
| 32 | Dog, India | A07-0438 |
| 33 | Mongoose, Puerto Rico | A04-2027 |
| 34 | Gray fox, Arizona, USA | A04- 0716 |
| 35 | Skunk, Wisconsin, USA | A04-0713 |
| 36 | Dog/coyote, Texas, USA | A04-2022 |
| 37 | Human/wolf, Russia | A07-0437 |
| 38 | Bat, Tennessee, USA | A07-0455 |
| 39 | Dog, India | A07-0443 |
| 40 | Bat, Tennessee, USA | A07-0452 |
| 41 | Cow, Sri Lanka | A07-0440 |
| 42 | Bat, Washington, USA | A04-0724 |
| 43 | Bat, Australia  (ABLV) | NA^%^ |
| 44 | Bat, Australia  (ABLV) | SM 4476 |
| 45 | ERA | A04-709 (Alabama, USA, 1935^&^) |
| 46 | European bat lyssavirus type 1 | A09-3484 |
| 47 | European bat lyssavirus type 2 | A03-4659 |
| 48 | Duvenhage | SM1027 (South Africa, 1970^&^) |
| 49 | EBLV 1 | A09-3485 |
| 50 | EBLV 2 | 2 A09-3483 |

*: Identification number used to record the year of the virus collection (e.g., A04-2027, collected in 2004 and the last four numbers are automatically generated by the CDC accessioning system).

^%^: There is no information for the year/date of the virus isolation.

^&^: Date of the virus collection.

**S1 Fig. Antibody production and cell growth results during stability testing of stable CHO cell lines producing NP-19-9 and 11B6.** (A) antibody titer during batch culture and (B) doubling time

**
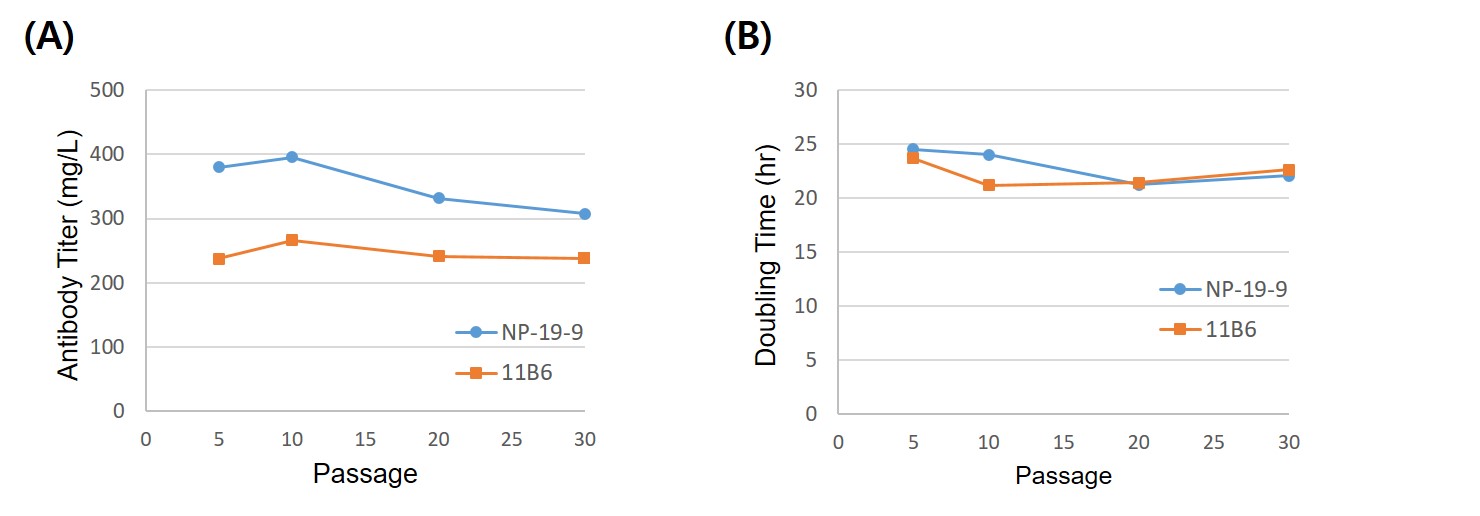
**

**S2 Table. Results of evaluation of neutralizing activity of antibody cocktail against various kinds of rabies viruses (RFFIT)**

| **No.** | **Virus** | **NP-19-9**  **Neutralizing activity (IU/mg)** | **11B6**  **Neutralizing activity (IU/mg)** | **Mixture (1:1)**  **Neutralizing activity (IU/mg)** |
| --- | --- | --- | --- | --- |
| 1 | Bat, New York, USA | 10714 | 2619 | 8571 |
| 2 | Bat, Pennsylvania, USA | 5263 | 5263 | 4421 |
| 3 | Coyote, Texas, USA | 5000 | 1647 | 2500 |
| 4 | Dog, India | 9412 | 3676 | 5000 |
| 5 | Dog, Gabon | 5313 | 2031 | 4531 |
| 6 | Cow, Sri Lanka | 2000 | 2160 | 2000 |
| 7 | Skunk, Wisconsin, USA | 2160 | 2240 | 2240 |
| 8 | Dog, China | 2321 | 2143 | 1964 |
| 9 | Dog, Thailand | 9286 | 2286 | 7857 |
| 10 | Human/dog, Philippines | 5185 | 1389 | 4630 |
| 11 | Mongoose, South Africa | 9143 | 7714 | 9143 |
| 12 | Bat, Washington, USA | 10000 | 8214 | 8929 |
| 13 | CVS-11 | 7353 | 2059 | 6765 |

**S3 Table. Rabies viruses isolated in India**

| **Virus abbreviation** | **Source** | **Virus isolation area** | **Identification number  or  Year of isolation** |
| --- | --- | --- | --- |
| SV1 | Dog | Kerala, India | 2011 |
| SV2 | Dog | Kerala, India | 11-793 |
| SV3 | Human | Karnataka, India | 10-1289 |
| SV4 | Human | Karnataka, India | 2010 |
| SV5 | Dog | Chennai, Tamil Nadu, India | 11-791 |
| SV6 | Dog | Chennai, Tamil Nadu, India | 2011 |

**S4 Table. The results of in vitro and vivo testing using six rabies viruses from India.**

1. **In vitro test (Neutralizing activity test)**

| **Antibody** | **SV1** | **SV2** | **SV3** | **SV4** | **SV5** | **SV6** |
| --- | --- | --- | --- | --- | --- | --- |
| **NP-19-09** | > 3000 IU/mg | > 3000 IU/mg | > 3000 IU/mg | > 3000 IU/mg | > 3000 IU/mg | > 3000 IU/mg |
| **11B6** | > 2000 IU/mg | > 2000 IU/mg | > 2000 IU/mg | > 2000 IU/mg | > 2000 IU/mg | > 2000 IU/mg |
| **Mix** | > 3000 IU/mg | > 3000 IU/mg | > 3000 IU/mg | > 3000 IU/mg | > 3000 IU/mg | > 3000 IU/mg |
| **Negative Control** | 0 | 0 | 0 | 0 | 0 | 0 |
| **Positive Control** | > 100 IU/mg | > 100 IU/mg | > 100 IU/mg | > 100 IU/mg | > 100 IU/mg | > 100 IU/mg |

1. **In vitro test (Survival rate) at DPI40**

| **Antibody** | **SV1** | **SV2** | **SV3** | **SV4** | **SV5** | **SV6** |
| --- | --- | --- | --- | --- | --- | --- |
| **NP-19-09** | 80 | 90 | 90 | 80 | 90 | 90 |
| **11B6** | 100 | 100 | 100 | 100 | 100 | 100 |
| **Mix** | 100 | 100 | 100 | 100 | 100 | 100 |
| **Negative Control** | 0 | 0 | 0 | 0 | 0 | 0 |
| **Positive Control** | 100 | 100 | 100 | 100 | 100 | 100 |

**S5 Table. Genotype and phylogroup classifications for the lyssaviruses.**

**
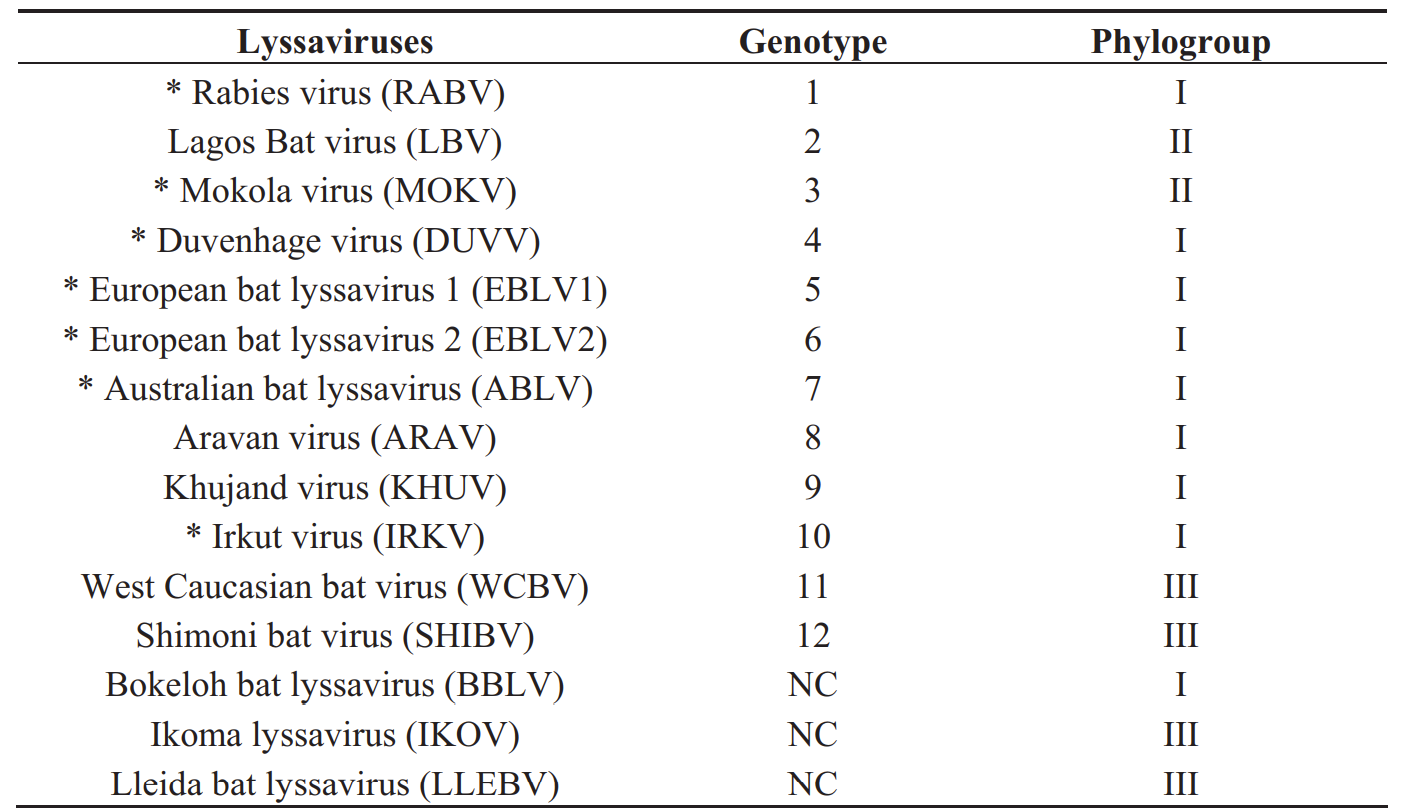
**

NC, not classified; these viruses are not yet classified as distinct lyssavirus species (genotypes) according to the International Committee on the Taxonomy of Viruses (ICTV) [34]. *, Human cases have been documented.
